# Supplementary material for: Modelling Growth and Decline in a Two-Species Model System: Pathogenic Escherichia coli O157:H7 and Psychrotrophic Spoilage Bacteria in Milk
Source: Foods. 2020 Mar 12;9(3):331. doi: 10.3390/foods9030331 (PMC7142549; doi:10.3390/foods9030331)

Table S1. Bayesian estimates of the posterior means and Highest Posterior Density intervals (HPD: 2.5 and 97.5%) of the growth (μ; day^{-1}) and decline (k; day^{-1}) rates of *E. coli* O157:H7 LCDC 86-51, *E. coli* O157:H7 ATCC 35150, non-pathogenic *E. coli*, and *P. fluorescens* cultured alone (EcO1, EcO2, Ec, Pf), or co-cultured (EcO1 + Pf, EcO2 + Pf, Ec + Pf) at 7, 13, 19 or 25 °C.

Cultures	Temp (ºC)	Strain	μ mean	HPD Intervals 2.5%	HPD Intervals 97.5%	k mean	HPD Intervals 2.5%	HPD Intervals 97.5%
EcO1, EcO2, Ec, Pf	7	EcO1	0.104	0.041	0.261	–	–	–
		EcO2	0.123	0.014	0.789	–	–	–
		Ec	0.120	– 0.019	1.382	–	–	–
		Pf	1.690	1.173	2.202	0.485	– 0.514	2.160
	13	EcO1	0.535	0.356	0.782	– 0.017	– 2.003	1.987
		EcO2	0.503	0.348	0.727	0.520	– 0.438	2.148
		Ec	0.494	0.345	0.688	0.007	– 1.937	1.965
		Pf	0.990	0.633	1.450	0.460	– 0.488	2.007
	19	EcO1	1.664	1.264	2.070	– 0.164	– 0.190	– 0.142
		EcO2	1.597	1.006	2.214	– 0.233	– 0.268	– 0.203
		Ec	1.577	0.919	2.232	– 0.212	– 0.240	– 0.187
		Pf	1.539	0.981	2.126	– 0.056	– 0.072	– 0.045
	25	EcO1	2.550	1.505	3.336	– 0.179	– 0.209	– 0.154
		EcO2	2.781	1.591	3.609	– 0.247	– 0.285	– 0.215
		Ec	2.007	1.714	2.275	– 0.170	– 0.194	– 0.150
		Pf	1.775	1.185	2.348	– 0.128	– 0.147	– 0.111
EcO1 + Pf	7	EcO1	0.563	0.235	0.918	– 0.281	– 0.447	0.538
		Pf	2.021	1.357	2.749	– 0.036	– 0.116	0.361
	13	EcO1	0.692	0.425	1.026	0.242	– 1.292	2.028
		Pf	0.944	0.670	1.218	0.256	– 1.247	2.005
	19	EcO1	1.596	1.138	2.020	– 1.804	– 2.467	– 1.264
		Pf	1.579	1.049	2.025	– 0.064	– 0.140	– 0.025
	25	EcO1	1.358	– 0.900	3.259	– 0.262	– 0.304	– 0.225
		Pf	1.167	– 0.869	2.628	– 0.131	– 0.157	– 0.108
EcO2 + Pf	7	EcO2	0.824	0.357	1.208	– 0.017	– 1.841	2.009
		Pf	2.006	1.406	2.645	0.202	– 1.108	1.952
	13	EcO2	0.664	0.420	0.952	0.130	– 1.293	2.017
		Pf	0.936	0.660	1.205	0.163	– 1.332	1.989
	19	EcO2	1.624	1.226	2.019	– 0.260	– 0.300	– 0.224
		Pf	1.641	1.239	2.047	– 0.046	– 0.062	– 0.030
	25	EcO2	1.857	0.187	2.791	– 1.371	– 2.210	– 0.304
		Pf	1.782	0.306	2.738	– 0.698	– 2.129	– 0.168
Ec + Pf	7	Ec	0.581	0.357	0.791	– 0.222	– 1.738	1.835
		Pf	1.679	0.855	2.227	– 0.019	– 1.174	1.856
	13	Ec	0.945	0.661	1.270	0.266	– 1.468	2.104
		Pf	1.204	0.854	1.487	0.319	– 1.305	2.078
	19	Ec	1.495	1.114	1.907	– 1.709	– 2.064	– 1.089
		Pf	1.606	1.185	2.030	– 0.072	– 0.156	– 0.038
	25	Ec	2.178	1.667	2.620	– 0.143	– 0.311	– 0.065
		Pf	2.575	1.957	3.149	– 0.095	– 0.128	– 0.066

Table S2. Bayesian estimates of the posterior means and Highest Posterior Density intervals (HPD: 2.5 and 97.5%) of the standard deviations for growth (σ) and decline (–σ) periods of *E. coli* O157:H7 LCDC 86-51, *E. coli* O157:H7 ATCC 35150, non-pathogenic *E. coli*, and *P. fluorescens* cultured alone (EcO1, EcO2, Ec, Pf), or co-cultured (EcO1 + Pf, EcO2 + Pf, Ec + Pf) at 7, 13, 19 or 25 °C.

**Cultures**	**Temp (°C)**	**Strain**	**σ mean**	**HPD Intervals**		**– σ mean**	**HPD Intervals**	
				**2.5%**	**97.5%**		**2.5%**	**97.5%**
EcO1, EcO2, Ec, Pf	7	EcO1	0.234	0.151	0.376	–	–	–
		EcO2	0.292	0.182	0.499	–	–	–
		Ec	0.388	0.240	0.666	–	–	–
		Pf	0.554	0.291	1.111	0.749	0.397	1.492
	13	EcO1	0.605	0.350	1.090	0.990	0.452	2.212
		EcO2	0.552	0.322	1.000	0.891	0.406	1.986
		Ec	0.528	0.308	0.932	1.107	0.469	2.239
		Pf	0.662	0.378	1.219	0.275	0.125	0.661
	19	EcO1	0.398	0.206	0.826	0.664	0.317	1.466
		EcO2	0.619	0.329	1.228	0.724	0.360	1.565
		Ec	0.658	0.346	1.302	0.621	0.302	1.352
		Pf	0.598	0.312	1.199	0.465	0.211	1.104
	25	EcO1	0.578	0.275	1.302	0.684	0.328	1.497
		EcO2	0.536	0.241	1.290	0.728	0.352	1.575
		Ec	0.210	0.104	0.460	0.563	0.271	1.258
		Pf	0.641	0.333	1.298	0.600	0.286	1.342
EcO1 + Pf	7	EcO1	0.447	0.247	0.860	0.485	0.173	1.493
		Pf	0.729	0.409	1.346	0.281	0.098	0.806
	13	EcO1	0.862	0.483	1.575	0.765	0.380	1.586
		Pf	0.492	0.260	0.979	0.283	0.130	0.672
	19	EcO1	0.581	0.291	1.181	1.930	1.056	3.555
		Pf	0.612	0.308	1.271	0.707	0.298	1.626
	25	EcO1	1.263	0.401	2.827	0.552	0.307	1.057
		Pf	0.935	0.354	2.165	0.542	0.301	1.027
EcO2 + Pf	7	EcO2	0.387	0.188	0.852	1.981	1.062	3.511
		Pf	0.646	0.340	1.288	1.296	0.674	2.395
	13	EcO2	0.747	0.415	1.367	0.770	0.274	1.817
		Pf	0.504	0.270	1.018	0.214	0.090	0.561
	19	EcO2	0.554	0.312	1.022	0.470	0.190	1.245
		Pf	0.553	0.311	1.029	0.414	0.160	1.107
	25	EcO2	0.986	0.378	2.530	1.419	0.736	2.612
		Pf	1.125	0.445	2.558	1.868	0.984	3.427
Ec + Pf	7	Ec	0.217	0.091	0.508	1.228	0.714	2.470
		Pf	0.645	0.297	1.524	1.695	0.816	3.205
	13	Ec	0.577	0.303	1.086	0.570	0.230	1.450
		Pf	0.372	0.174	0.825	0.130	0.044	0.407
	19	Ec	0.588	0.333	1.103	1.764	0.915	3.434
		Pf	0.562	0.312	1.061	0.449	0.130	1.335
	25	Ec	0.415	0.213	0.819	1.750	0.885	3.549
		Pf	0.760	0.443	1.354	0.653	0.270	1.637

Table S3. Maximal population density (N_{max}; log CFU/mL) and time to reach (ttr; day) a population density of 6 or 8 log CFU/mL of *E. coli* spp. strains and *P. fluorescens* cultured alone or in co-culture at 7, 13, 19, or 25 °C.

Temp (°C)	Cultures	N_{max}	Time to Reach (ttr) a Population of	
			6 log CFU/mL	8 log CFU/mL
7	EcO1	4.41	–	–
	EcO2	4.42	–	–
	Ec	4.80	–	–
	Pf	8.76	0.72	0.96
	EcO1(Pf)	5.30	–	–
	EcO2(Pf)	5.37	–	–
	Ec(Pf)	5.20	–	–
	Pf(EcO1)	8.73	0.56	1.04
	Pf(EcO2)	8.79	0.56	1.04
	Pf(Ec)	9.01	0.56	1.12
13	EcO1	8.46	2.08	3.36
	EcO2	8.33	2.08	3.52
	Ec	8.25	2.24	3.68
	Pf	8.28	1.12	1.76
	EcO1(Pf)	8.29	2.24	3.52
	EcO2(Pf)	8.29	2.08	3.52
	Ec(Pf)	8.20	1.60	3.68
	Pf(EcO1)	8.33	0.96	1.76
	Pf(EcO2)	8.30	0.96	1.76
	Pf(Ec)	8.21	0.64	1.44
19	EcO1	8.37	0.56	1.04
	EcO2	8.17	0.64	1.04
	Ec	8.04	0.64	1.04
	Pf	8.11	0.64	1.04
	EcO1(Pf)	8.26	0.56	1.04
	EcO2(Pf)	8.23	0.56	1.04
	Ec(Pf)	7.89	0.64	1.12
	Pf(EcO1)	8.14	0.56	0.96
	Pf(EcO2)	8.11	0.56	1.04
	Pf(Ec)	8.13	0.56	1.04
25	EcO1	8.22	0.32	0.64
	EcO2	8.33	0.32	0.64
	Ec	8.28	0.40	0.96
	Pf	8.94	0.48	0.88
	EcO1(Pf)	8.43	0.32	0.56
	EcO2(Pf)	8.54	0.40	0.56
	Ec(Pf)	8.10	0.36	0.80
	Pf(EcO1)	8.71	0.48	0.72
	Pf(EcO2)	8.98	0.40	0.64
	Pf(Ec)	9.08	0.40	0.56

Figure S1. Hamiltonian Monte Carlo Method (HMCM) diagnosis plots of the growth rates of *E. coli* O157:H7 LCDC 86-51 (EcO1), *E. coli* O157:H7 ATCC 35150 (EcO2) or *E. coli* ATCC 8739 (Ec) co-cultured with *P. fluorescens* (EcO1(Pf), EcO2(Pf), or Ec(Pf)) at 7 °C. Panels show three plots for the growth rate parameter: traces or value estimated in each step of the HMM (left); the parameter posterior distributions (middle); and the autocorrelation functions for the parameter estimates (right). mu[1] and mu[2] are the growth rates of *E. coli* spp or *P. fluorescens*, respectively.

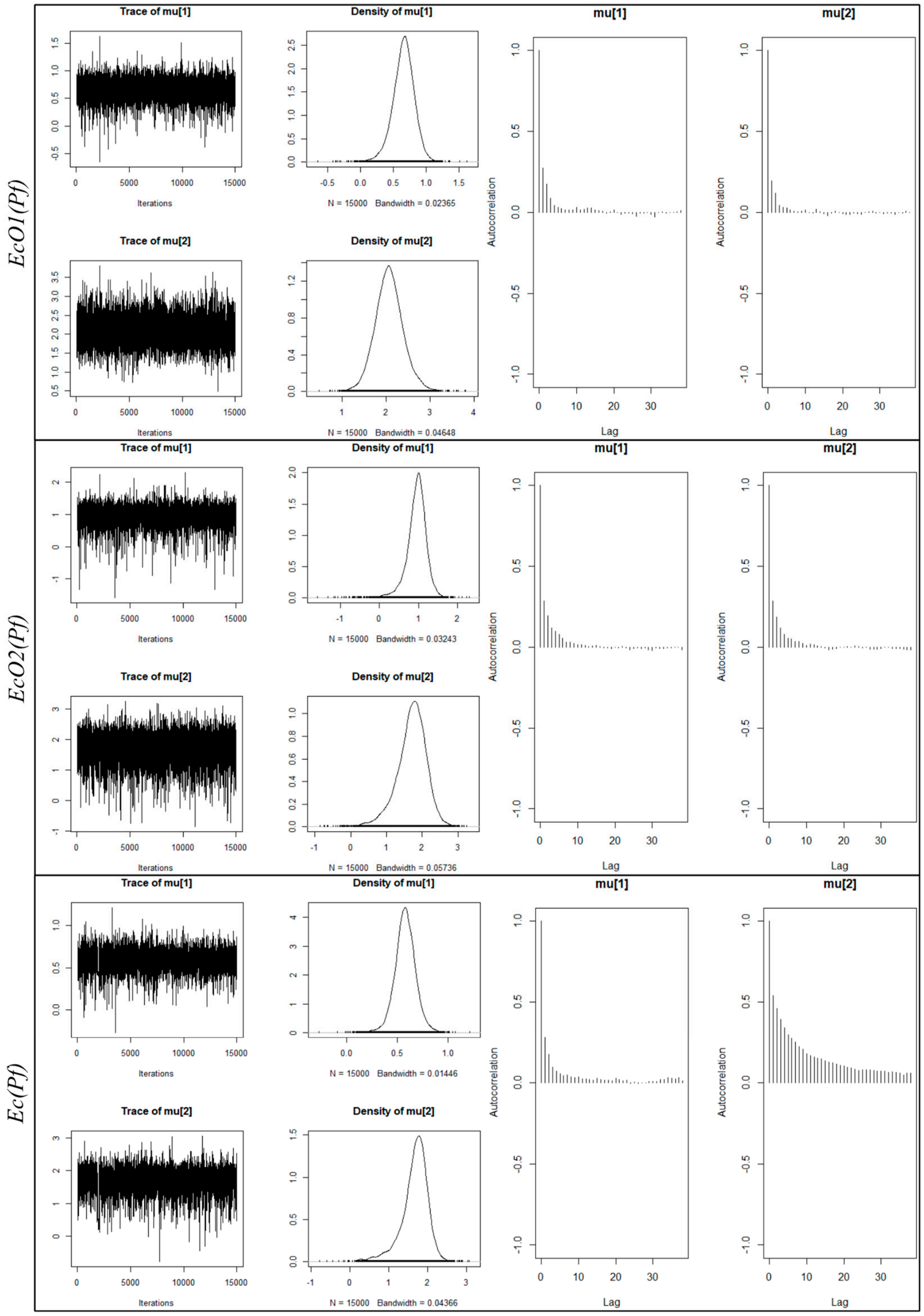

Figure S2. Hamiltonian Monte Carlo Method (HMCM) diagnosis plots of the decline rates of *E. coli* O157:H7 LCDC 86-51 (EcO1), *E. coli* O157:H7 ATCC 35150 (EcO2) or *E. coli* ATCC 8739 (Ec) co-cultured with *P. fluorescens* at 7 °C. See legends and explanations in Figure S1. k[1] and k[2] are the decline rates of *E. coli* spp or *P. fluorescens*, respectively.

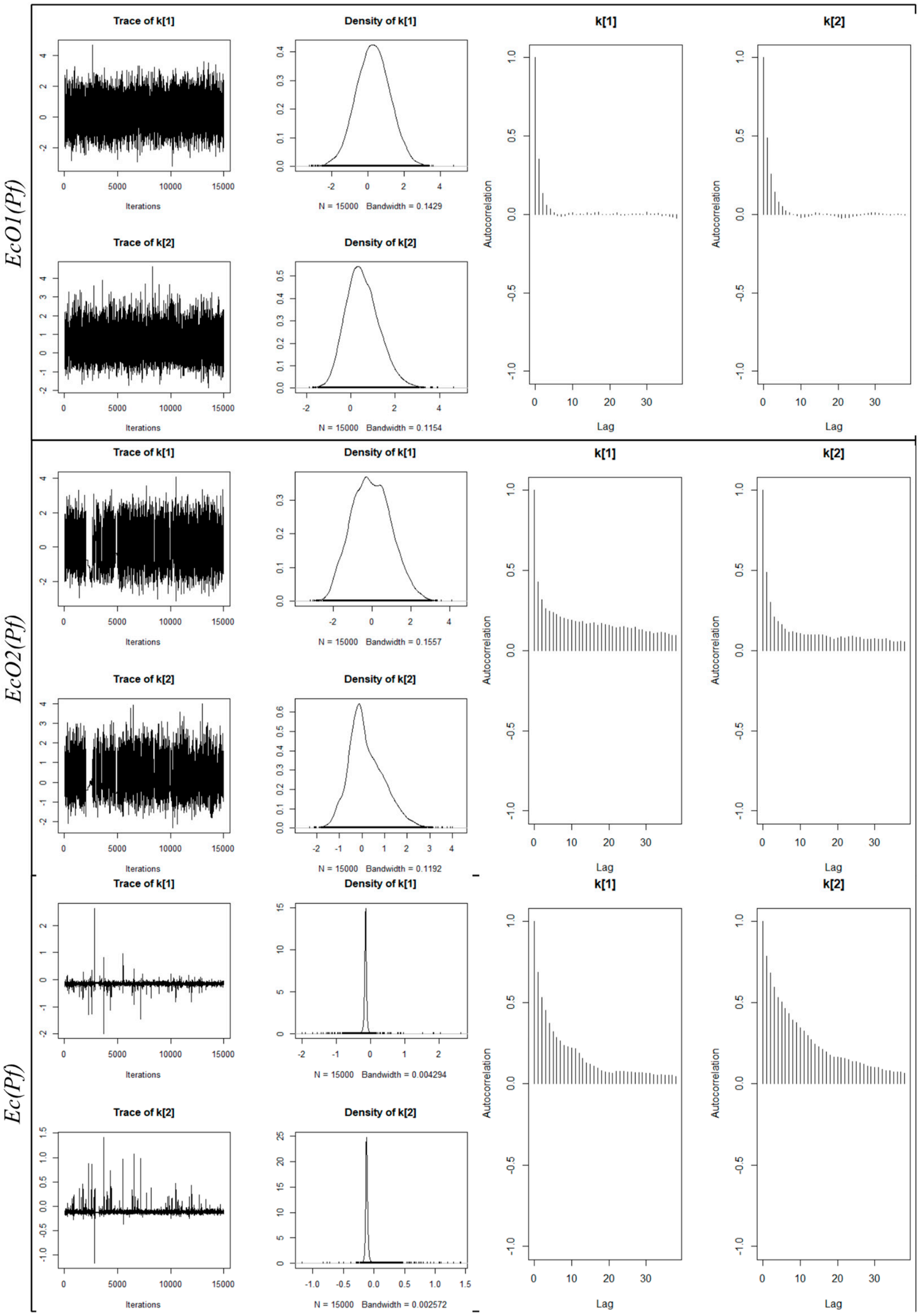

Figure S3. Hamiltonian Monte Carlo Method (HMCM) diagnosis plots of the growth rates of *E. coli* O157:H7 LCDC 86-51 (EcO1), *E. coli* O157:H7 ATCC 35150 (EcO2) or *E. coli* ATCC 8739 (Ec) co-cultured with *P. fluorescens* at 25 °C. See legends and explanations in Figure S1.

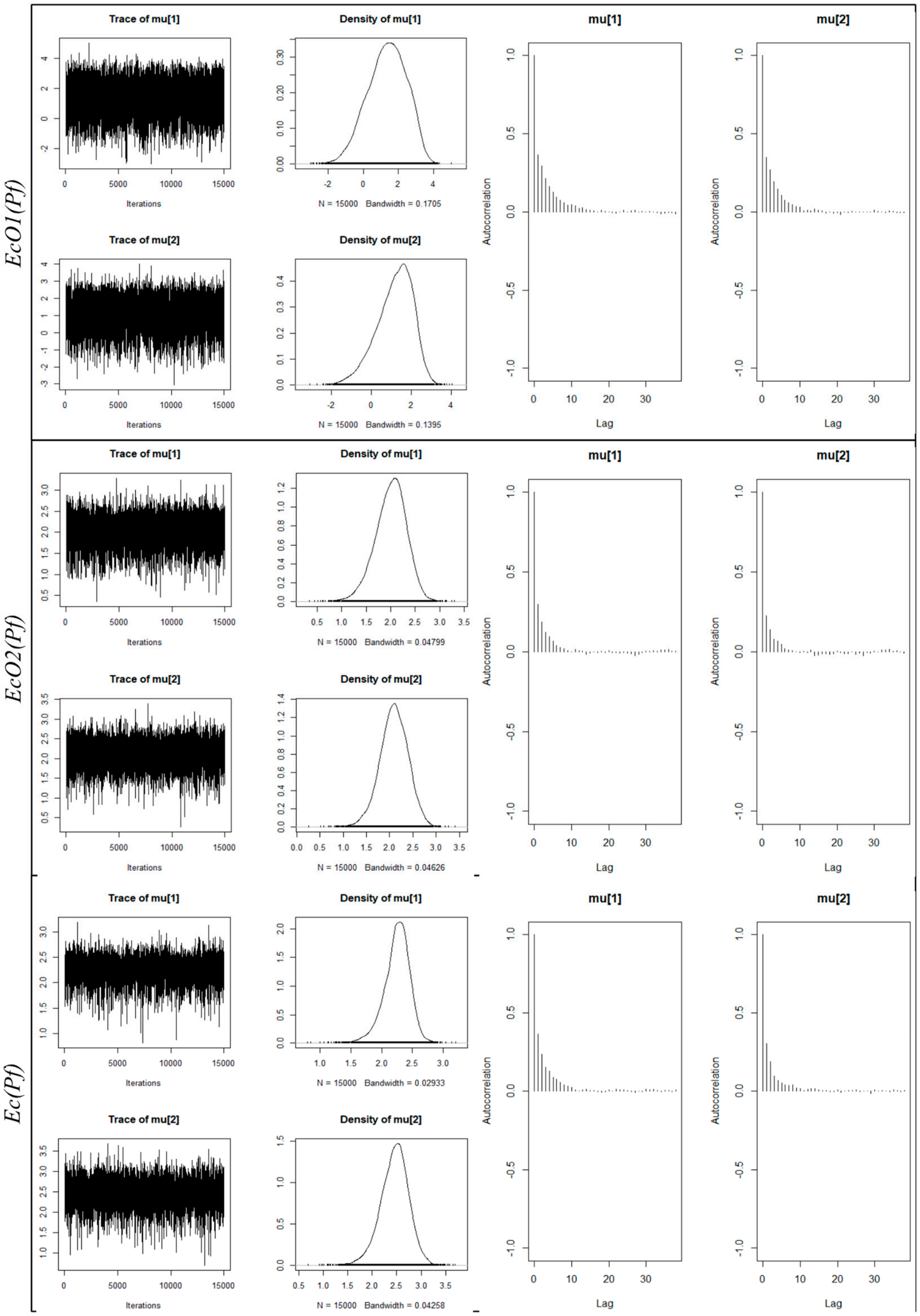

Figure S4. Hamiltonian Monte Carlo Method (HMCM) diagnosis plots of the decline rates of *E. coli* O157:H7 LCDC 86-51 (EcO1), *E. coli* O157:H7 ATCC 35150 (EcO2) or *E. coli* ATCC 8739 (Ec) co-cultured with *P. fluorescens* at 25 °C. See legends and explanations in Figures S1 and S2.

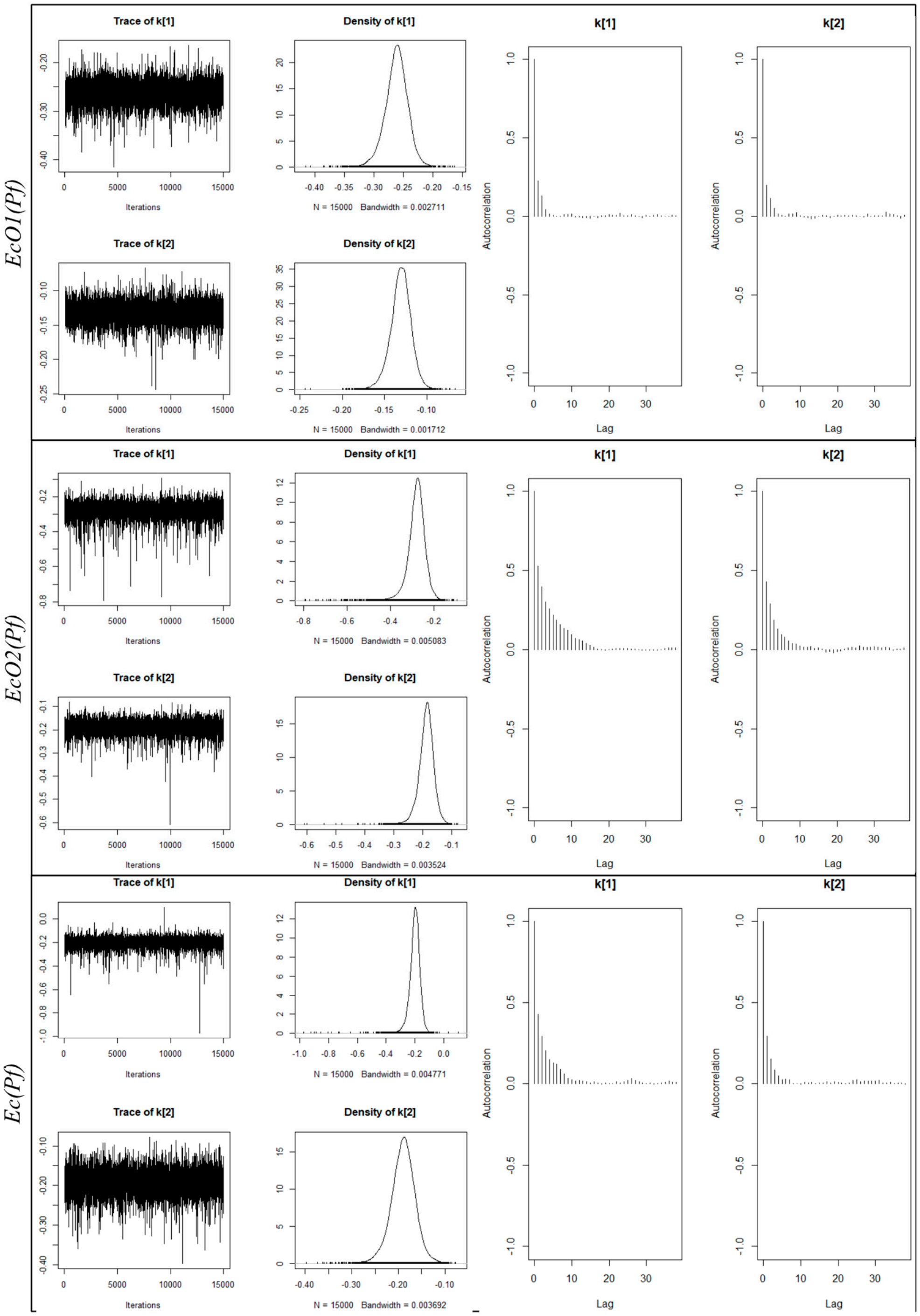

Supplement: Supplementary file 1 [file foods-09-00331-s001.pdf]
